# Contrast enhancement imaging in coronary arteries in patients with systemic lupus erythematosus

**DOI:** 10.1186/1532-429X-14-S1-P178

**Published:** 2012-02-01

**Authors:** Valentina O Puntmann, David D'Cruz, Peter Taylor, Tarique Hussain, Andreas Indermuehle, Britta Butzbach, Rene M Botnar, Eike Nagel

**Affiliations:** 1 Cardiovascular Imaging, King's College London, London, UK; 2 The Lupus Unit, King's College London, London, UK; 3 Nuffield Department of Orthopaedics, Rheumatology&Musculoskeletal Sciences, University of Oxford, Oxford, UK

## Summary

Patients with SLE suffer from accelerated atherosclerosis due to systemic inflammation. We examined subclinical coronary artery involvement in patients with systemic lupus erythematous (SLE) by contrast enhanced inversion recovery (CE-IR) coronary magnetic resonance imaging.

## Background

Vessel wall inflammation plays a key role in the initiation and progression of atherosclerosis, from endothelial injury to remodeling and plaque formation. Cardiovascular (CV) magnetic resonance (CMR) provides noninvasive visualization and characterization of arterial remodeling both in the great vessels, and also the coronary arteries. Contrast-enhanced inversion-recovery (CE-IR) prepared coronary imaging allows detection of vessel wall enhancement by visualization of contrast agent uptake. We examined subclinical coronary artery involvement in patients with systemic lupus erythematous (SLE) by contrast enhanced inversion recovery (CE-IR) coronary magnetic resonance imaging.

## Methods

In 19 SLE patients in stable remission (male, n=4), we performed CE-IR magnetic resonance coronary imaging 40 minutes after administration of gadolinium contrast agents. Contrast-to-noise ratio (CNR) within the coronary artery and ascending aortic vessel wall was quantified and compared to age and gender-matched apparently healthy controls (n=9).

## Results

Results. There was a significant increase in mean coronary CNR in SLE patients (SLE vs. controls: 7.7±2 vs. 3.9±0.9, p<0.01). CNR within aortic wall was significantly raised in the SLE group (11.2±2.4 vs. 6.8± 1.9, p=0.008). In SLE patients, coronary CNR correlated with history of antiphospholipid syndrome (APS) (r=0.73, p=0.003), duration of disease (r=0.63, p=0.04) and ESR (r=0.54, p=0.04). In the SLE group, multivariate linear regression identified APS as independent predictor of coronary CNR (R2=0.26, F=11.2, p<0.001), whereas systolic blood pressure showed association with aortic CNR (R2=0.23 F=5.4, p=0.01). ROC analysis to discriminate healthy controls from the SLE group by increased coronary CNR revealed an optimal cut-off of 5.2 with 100% sensitivity and specificity.

## Conclusions

Our study for the first time demonstrates subclinical coronary artery involvement in patients with SLE and history of systemic inflammation. These findings are associated with markers of inflammation, more aggressive subtype and duration of disease, but are independent of traditional CV risk factors.

## Funding

NIHR BRC (Atherosclerosis theme).

**Figure 1:**
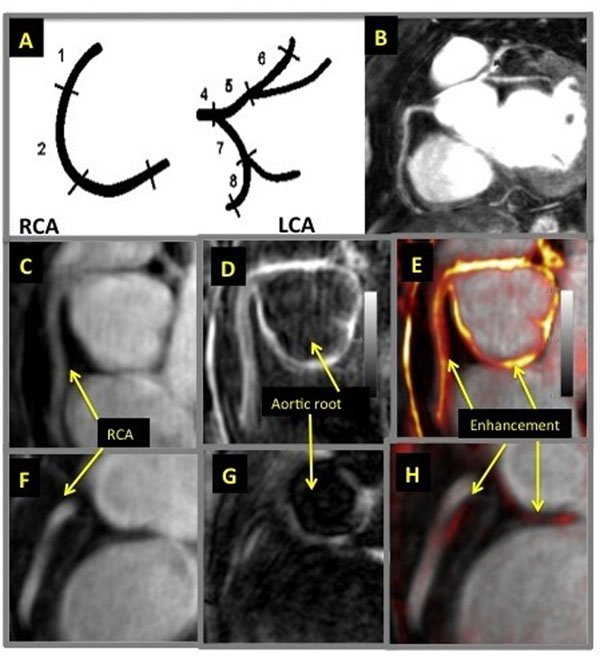
Figure 1. A and B - anatomical and morphological relationships following segmental model of epicardial coronary artery tree. The right coronary artery (RCA) was analyzed in 3 segments (1, 2, and 3), the left coronary artery (LCA) within the left main stem (4), the left anterior descending (5 and 6), and the circumflex artery (7 and 8). C-E: representative images of RCA from a 38-year old female patient with SLE and F-H from age/gender matched healthy subject. C and F - CMR coronary angiography with luminogram, used to measure vessel length and lumen diameter. D and G -IR-CE images with black-blood prepulse to null the blood signal and reveal enhancement within aortic and coronary vessel wall. E and H - fused images of both previous ones to depict enhancement (orange) in relation to vessel lumen (bright signal).

